# The Distribution of Respiratory Viral Pathogens Among the Symptomatic Respiratory Tract Infection Patients From Dhaka City in the Pre-COVID-19 Pandemic Era

**DOI:** 10.7759/cureus.70781

**Published:** 2024-10-03

**Authors:** SM Rashed Ul Islam, Asish Kumar Ghosh, Mst. Nurjahan Begum, Mohammad Shahjahan Siddike Shakil, Munira Jahan, AK Qumrul Huda

**Affiliations:** 1 Virology, Bangabandhu Sheikh Mujib Medical University (BSMMU), Dhaka, BGD; 2 Virology, Dhaka Medical College, Dhaka, BGD; 3 Anesthesia, Analgesia, and Intensive Care Medicine, Bangabandhu Sheikh Mujib Medical University (BSMMU), Dhaka, BGD; 4 Respiratory Medicine, National Institute of Diseases of the Chest and Hospital, Dhaka, BGD

**Keywords:** medical intensive care unit (micu), multiplex pcr system, pre-covid-19, respiratory tract infections, respiratory virus

## Abstract

Rapid and accurate diagnosis is crucial for determining the etiology and, perhaps, effectively treating and preventing viral respiratory infections. A multiplex quantitative reverse transcription polymerase chain reaction (qRT-PCR) assay⁠⁠⁠⁠⁠⁠⁠ was utilized to determine the prevalence of viral etiology in cases of acute respiratory tract infections (ARTIs). Outpatient department (OPD) and intensive care unit (ICU) patients with fever and respiratory symptoms were enrolled from December 2018 to April 2020. Nucleic acids were extracted from the respiratory tract samples using the SV Total RNA Isolation System (Promega Corporation, Madison, WI), and virus identification was performed using qRT-PCR assay (Fast Track Diagnostics {FTD} Respiratory Pathogens, Esch-sur-Alzette, Luxembourg). A total of 152 samples were collected from OPD and ICU. Among them, 32.23% (n = 49) of the patients were positive for at least one respiratory virus. From 49 infected cases, 42 had only a single viral pathogen, whereas seven had co-infections. Of the patients, 32.25% (30) in the OPD and 32.20% (19) in the ICU tested positive for the respiratory viral pathogen. Among the OPD patients, human coronaviruses (HCoVs) OC43, 229E, NL63, and HKU1 were detected as predominant viruses (10.75%), followed by influenza virus (IFV) (8.6%), human rhinoviruses (HRVs) (6.45%), human parainfluenza viruses (HPIVs) (6.45%), respiratory syncytial virus (RSV) (3.22%), and adenovirus (2.15%). In ICU cases, HPIV and HRV were detected as predominant viruses (8.47% each), followed by HCoV (5.08%), human metapneumovirus (HMPV) (5.08%), influenza A virus (IAV) (3.38%), adenovirus (3.38%), and RSV (1.69%). This study highlighted the prevalence of respiratory viruses in both the community and hospital settings during pre-COVID-19, indicating a significant presence among patients in these environments.

## Introduction

Acute respiratory tract infections (ARTIs) are a major cause of illness and death worldwide [1]. They comprise upper respiratory tract infections (RTIs) presented as laryngitis, acute rhinosinusitis, pharyngitis, and acute otitis media and lower RTIs presented as bronchitis, pneumonia, and bronchiolitis [2]. ARTIs are responsible for almost 30% of deaths among children under five years of age worldwide, with a 2019 global burden study reporting 33 million episodes of ARTI, leading to 3.6 million hospitalizations and 26,300 in-hospital deaths in this age group [3]. In Bangladesh, 55% of children below the age of five and 40% of children exhibiting symptoms indicative of ARTIs sought medical care from healthcare facilities [4]. An approximate significant burden of community-acquired pneumonia (CAP) within the adult population of the United States, necessitating hospitalization, exceeded 1.5 million cases, with a mortality rate of 6.5% observed among this demographic [5]. The etiology of respiratory tract infections is predominantly viruses, with the highest cases reported from India (43 million), China (21 million), Pakistan (10 million), Bangladesh (6.4 million), Indonesia (six million), and Nigeria (6.1 million) [6]. Clinical manifestations of ARTI include cough, fever, chest pain, tachypnea, and sputum production. Upper RTIs (URTIs) present with symptoms such as cough, sore throat, runny nose, nasal congestion, headache, low-grade fever, facial pressure, sneezing, and malaise, which usually resolve on their own. Lower RTIs (LRTIs) share similar symptoms but can also involve severe cough, fever, rapid or difficult breathing, wheezing, skin turning blue, and chest pain [7]. The clinical manifestations of most RTIs overlap in various aspects, rendering the conclusive diagnosis of such conditions highly challenging [8]. The humid climate of Bangladesh is particularly inducive to the dispersal of such viruses, resulting in respiratory viruses causing approximately 60% of acute lower respiratory infections (ALRIs) [9]. The most common viruses associated with respiratory tract infections (RTIs) in adults are the influenza virus (IFV), accounting for 19.3% of RTIs, followed by human rhinovirus (HRV) at 6.5%, parainfluenza virus (PIV) at 4.3%, enterovirus (EV) at 3.2%, and human coronavirus (HCoV) at 1.1%, with adenoviruses (ADVs), respiratory syncytial viruses (RSVs), and human metapneumoviruses (HMPVs) also reported as pathogens, though HMPVs have rarely been detected in adults, showing a positivity rate of less than 1% [10].

ARTIs pose a serious public health burden in Bangladesh, particularly in densely populated urban areas such as Dhaka. The most attributable deaths were among children under five years old and those over 80 years of age. Pneumococci, followed by RSV and influenza, were identified as the largest etiological causes [11]. A Global Burden of Disease (GBD) study in 2016 found that pneumococcus and RSV were responsible for 50.1% and 3.2% of the attributable deaths among all ages in 2016, respectively. In children younger than five years and adults above 70 years, Hib (7.4%) and influenza (2.3%) were found to be the second and third most common causes, respectively [12].

This study was conducted to provide a detailed analysis of the prevalence and types of respiratory viruses circulating in Dhaka, Bangladesh. Understanding the viral landscape is crucial for improving the diagnosis, management, and treatment of ARTIs in this region. This study addresses a significant gap in local epidemiological data on respiratory viruses in urban Bangladesh, where the burden of ARTIs is high and often associated with severe outcomes. The objective of this study was to detect respiratory viruses in the population residing in Dhaka City, including both community and hospital set up during the pre-COVID-19 pandemic era.

## Materials and methods

Subject enrollment and sampling

The cross-sectional study population consisted of 152 patients aged eight years and above with clinical features suggesting acute respiratory tract infections from December 2018 to April 2020. Samples taken from patients seen in the outpatient department (OPD) of the National Institute of Diseases of the Chest and Hospital (NIDCH) were designated as community samples, while samples taken from the intensive care unit (ICU) of Bangabandhu Sheikh Mujib Medical University (BSMMU)'s Department of Anesthesia, Analgesia, and Intensive Care Medicine were designated as hospital samples. The sample size was determined according to the prevalence rate of the respiratory viral pathogen from the previous article [13].

A total of 251 participants were initially recruited to determine the appropriate sample size for the research. The pilot study and preliminary analysis indicated that a sample size of 150 would provide sufficient statistical power to achieve the study's objectives. In determining the appropriate sample size, a standard equation was used: n = N. Z2. p(1 - p)/E2. (N - 1) + Z2. p(1 - p), where n = required sample size, N = total population, Z = Z-score (standard normal deviation, which corresponds to the confidence level, usually 1.96 for a 95% confidence interval {CI}), P = estimated prevalence or proportion of the condition in the population (from previous studies or pilot data), and E = margin of error (precision level, often set at 5% or 0.05).

The feasibility of obtaining adequate respiratory samples from symptomatic patients within a reasonable timeframe was also considered. A larger sample size may require more resources, which limits the study.

The patients were enrolled according to criteria such as cough, fever, sneezing, running nose, and lung consolidation in chest X-rays. Patients aged eight years and older with symptoms such as cough, fever, and lung consolidation were included. The exclusion criteria involved patients with non-respiratory conditions or inadequate sample collection.

Sample collection techniques and storage

The respiratory samples included sputum and oropharyngeal swabs from community samples and tracheal aspirates from the ICU samples. Specimens were collected in phosphate-buffered saline (PBS) in a sterile container at 4°C. Next, it was transported to the Department of Virology at BSMMU for further laboratory procedures. During this process, the healthcare providers were instructed to use the World Health Organization (WHO) standard personal protective equipment (PPE).

Laboratory methods

Specimen Processing and Viral RNA Extraction

Specimen liquefaction was done by heating the samples at 60°C for 15 minutes. Then, the nucleic acid was extracted from the liquefaction sample using the SV Total RNA Isolation System (Promega Corporation, Madison, WI) per the manufacturer's instructions. In summary, this methodology integrates the disruptive and protective characteristics of guanidine thiocyanate (GTC) alongside β-mercaptoethanol to effectively inactivate the ribonucleases that are present within the samples. GTC, when combined with sodium dodecyl sulfate (SDS), functions to disrupt nucleoprotein complexes, thereby facilitating the liberation of RNA into solution and enabling its isolation free from protein contaminants. Following centrifugation to remove precipitated proteins and cellular debris from the lysate, the RNA undergoes selective precipitation utilizing ethanol and subsequently binds to the silica surface of the glass fibers located within the spin basket. By efficiently eliminating precipitated proteins and cellular debris from the lysate, these clarified lysates can be affixed to the spin baskets via a centrifugation technique. The binding interaction transpires promptly owing to the disruption of water molecules induced by the chaotropic salts, thereby promoting the adsorption of nucleic acids onto the silica. Ribonuclease (RNase)-free deoxyribonuclease (DNase) I is directly administered onto the silica membrane to degrade any contaminating genomic DNA. The total RNA that is bound is subsequently purified from residual salts, proteins, and cellular impurities through a series of straightforward washing procedures. Ultimately, the total RNA is eluted from the membrane through the introduction of nuclease-free water. The eluted RNA is now prepared for subsequent analytical procedures.

Molecular Detection

Utilizing an Applied Biosystems 7500 Real-Time PCR System from Thermo Fischer Scientific (Waltham, MA), respiratory viruses were identified through a multiplex real-time polymerase chain reaction (PCR) assay with Fast Track Diagnostics (FTD) multiplex real-time reverse transcription (RT)-PCR from Fast Track Diagnostics in Esch-sur-Alzette, Luxembourg. This PCR kit detects 16 viruses, such as influenza A virus (IAV); human rhinovirus (HRV); influenza B virus (IAV); human coronaviruses NL63, 229E, OC43, and HKU1; human parainfluenza viruses 2, 3, and 4; human bocavirus (HBoV); human metapneumovirus; respiratory syncytial virus (A/B); human adenovirus (HAdV); enterovirus (EV); and human parechovirus (HPeV). This kit encompasses three tube sets containing differentially labeled primer-fluorogenic probe mixtures, which are utilized for the detection of 16 viral RNA entities alongside *Streptococcus equi* employed as an internal control (IC) to assure a successful extraction and to exclude PCR inhibition. Additionally, it incorporates a plasmid control pool aimed at facilitating the identification of IAV, IBV, HRV, HCoV NL63/229E/OC43/HKU1, HPIV1-4, HMPV, human RSV (HRSV), and HAdV, serving as a positive control. Here, viral genomic RNA undergoes transcription into complementary DNA (cDNA) through a specific primer-mediated reverse transcription process, which is subsequently followed by immediate amplification within the same tube via polymerase chain reaction. The detection of specific target viral sequences within the reaction occurs through a measurable increase in fluorescence emitted by the corresponding dual-labeled probes, which is quantified as a cycle threshold (Ct) value by the real-time thermocycler. A positive test result is defined by the presence of an exponential trace that intersects the threshold cycle within 40 cycles. The Ct values of positive, negative, and internal controls serve as indicators of experimental validity and facilitate the monitoring of assay performance. The instructions delineated in the user's manual were strictly adhered to during the execution of laboratory protocols, and the results were evaluated accordingly.

Ethical approval

The study was approved by the Institutional Review Board (IRB) of BSMMU (BSMMU/2017/12853; 24/12/2017) per the guidelines for protecting human subjects. The participants or their guardians provided written informed consent after being briefed about the study.

Statistical analysis

Statistical comparisons between patient groups were evaluated using nonparametric methods, including the Mann-Whitney sum rank test for continuous variables and chi-squared analysis for categorical variables. Continuous variables were presented as mean ± SD, while categorical variables were reported as numbers (n) and percentages (%). A logistic regression analysis was conducted to estimate the odds ratios (OR) and 95% confidence intervals (CI) for each clinical feature, assessing the likelihood of testing positive for respiratory viruses. The clinical features included in this model were fever, cough, dyspnea, rhinorrhea, and wheezing. The association between variables was assessed using Fisher's exact test and Pearson's chi-squared test. A P value of <0.05 is considered as significant. All statistical analyses were conducted using the Statistical Package for Social Sciences (SPSS) software version 23.0 (IBM SPSS Statistics, Armonk, NY).

## Results

Demography and clinical profile of the study population

This research detected no statistically meaningful difference between the genders with respiratory virus infections. Among the patients infected by one or more respiratory pathogens, 67.35% were male, and 32.65% were female. Furthermore, no remarkable distinctions were observed in terms of the viruses infecting individuals of different genders. OPD patients reported a high rate of cough presentations; however, no significant difference was observed between positive and negative patients; those exposed to respiratory pathogens had higher rates of fever (p = 0.043), dyspnea (p = 0.007), rhinorrhea (p = 0.001), and wheezing (p = 0.043) than those whose pathogens were not detected.

Detection of respiratory viruses in clinical samples by multiplex PCR assay

Among 152 respiratory samples, 93 samples were collected from the community population (attended at OPD), and the rest (n = 59) were collected from severely ill patients from the hospital ICU. In this study, 49 (32.24%) samples were positive for at least one virus, and the rest, 103 (67.76%), were negative for any respiratory viruses as per multiplex PCR assay. Of the 49 positive samples, 42 (85.71%) were positive for only one virus, while seven (14.28%) were positive for two viruses. Among the 93 OPD samples, 30 (32.25%) were positive for respiratory viruses, and from 59 ICU samples, 19 (32.20%) were positive. The most frequently detected respiratory virus in OPD was HCoVs (10/30, 33.3%) and HPIV and rhinoviruses (RVs) (5/19, 26.31%) in ICU. In community samples, HCoVs and the influenza A virus (IAV) were found to be present at remarkably high frequencies, whereas in hospitalized patients, higher detection rates of HPIV and human RVs were observed.

**Figure 1:**
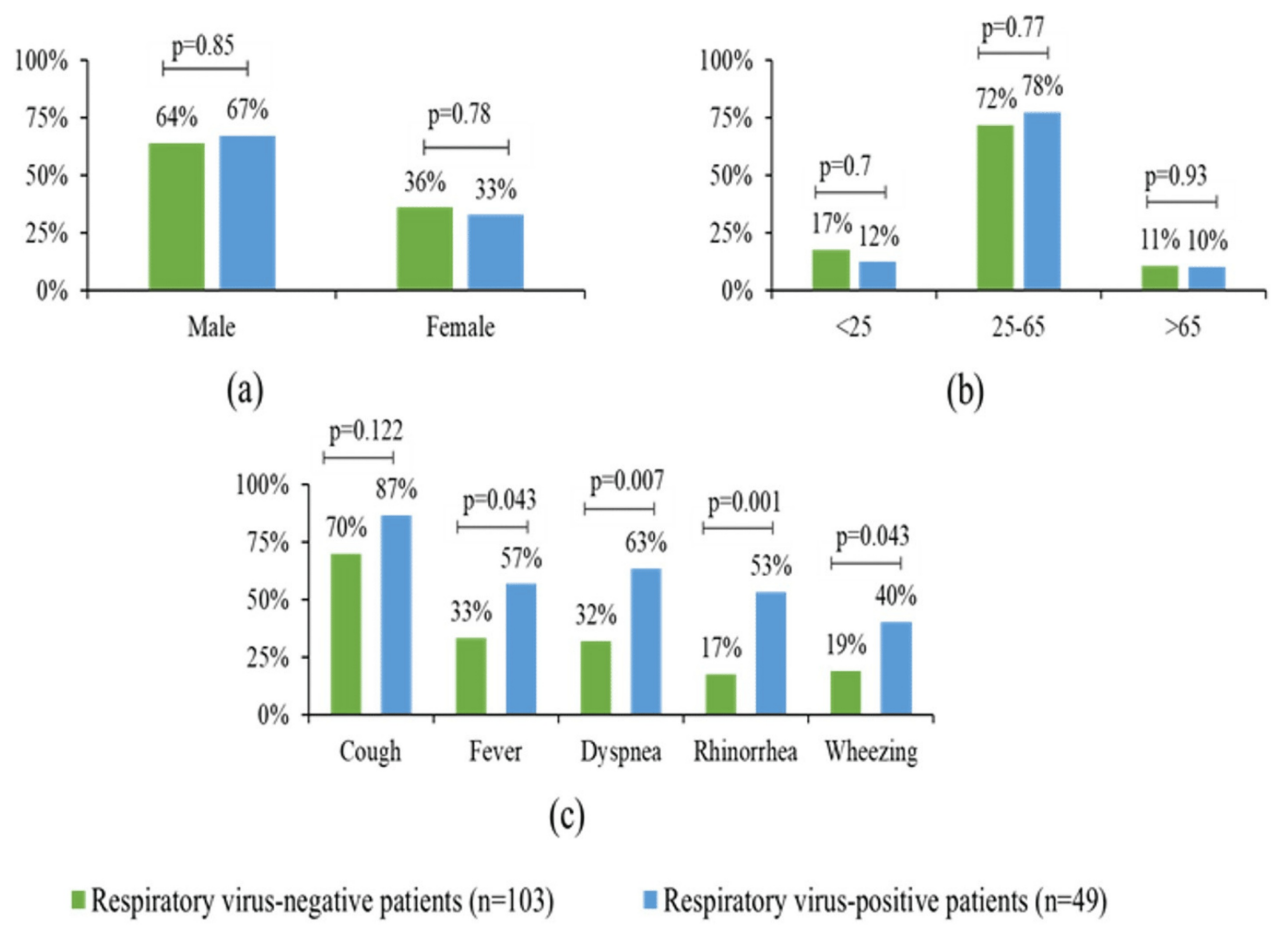
The patient demographics (a and b) and clinical findings (c) based on the study population's positive respiratory virus test results (p < 0.05)

The risk analysis revealed that patients presenting with fever had a significantly higher likelihood of testing positive for respiratory viruses (OR = 1.76, 95% CI = 1.05-2.93, and p = 0.043). Similarly, dyspnea (OR = 2.23, 95% CI = 1.24-4.01, and p = 0.007), rhinorrhea (OR = 2.89, 95% CI = 1.53-5.45, and p = 0.001), and wheezing (OR = 1.95, 95% CI = 1.02-3.72, and p = 0.043) were found to be associated with a higher risk of viral infection. However, no significant association was observed for cough (OR = 0.98, 95% CI = 0.59-1.63, and p = 0.938). These findings suggest that certain clinical features, such as dyspnea and rhinorrhea, are more predictive of viral infections in this population, while others, such as cough, are less indicative. Figure 1 displays the patient demographics and clinical results based on the respiratory viruses that were positive.

Co-detection of respiratory viruses

**Figure 2:**
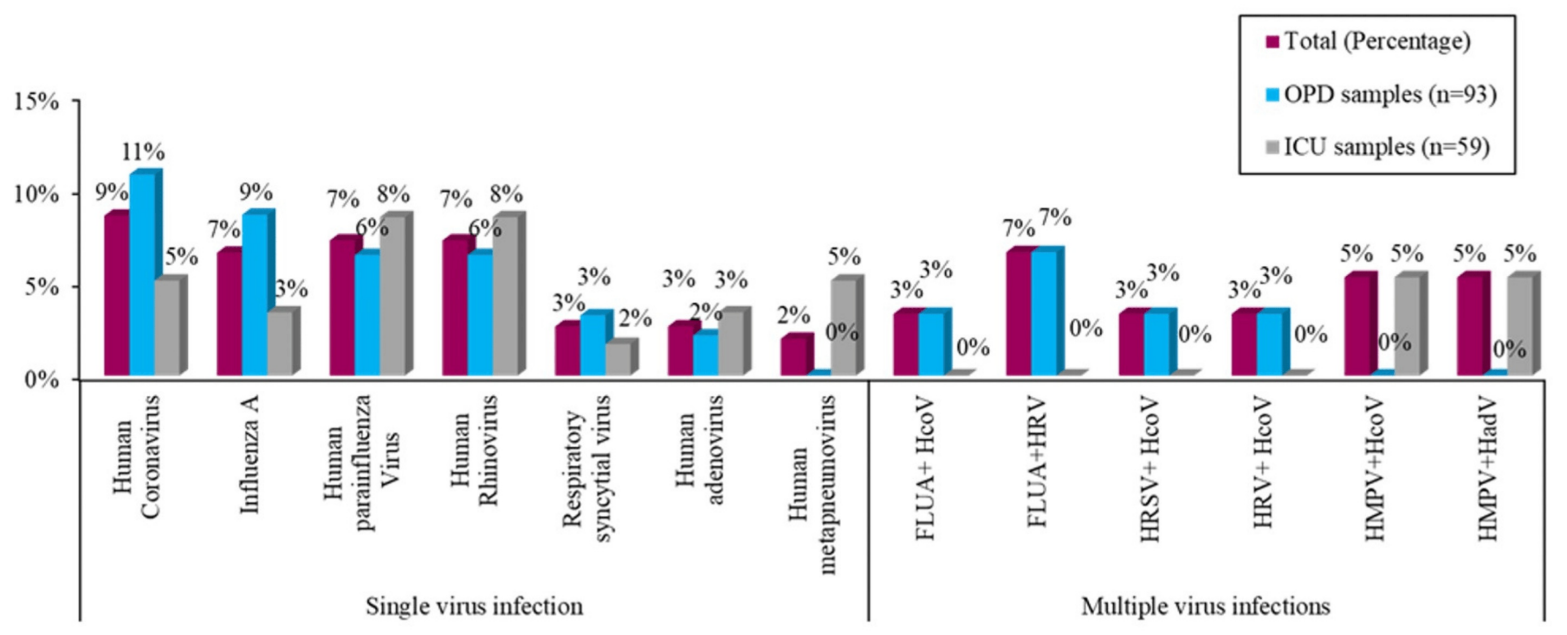
Distribution of respiratory viruses among the samples from the study population of OPD and ICU OPD, outpatient department; ICU, intensive care unit; HRSV, human respiratory syncytial virus

Of the 49 identified positive samples, 42 (85.71%) were positive for only one virus, as mentioned earlier, and seven positive cases (14.28%) had two respiratory viruses present simultaneously. Of those seven patients, four (HCoVs in particular) showed co-infection with multiple (dual) agents. Notably, IAV and HRVs were co-detected in three instances. The single virus infection and multiple virus infections are depicted in Figure 2.

**Figure 3:**
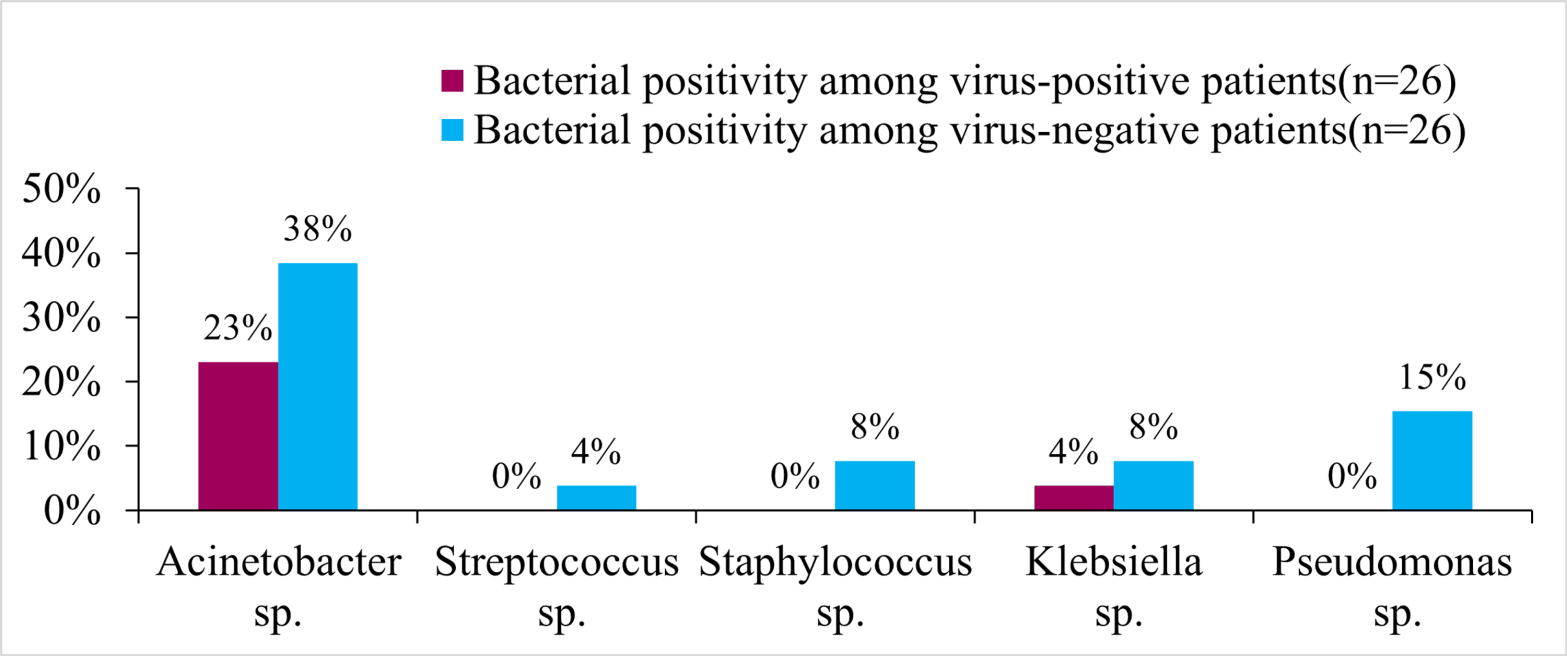
Distribution of bacterial culture-positive samples from the ICU based on the detectability of respiratory viruses ICU: intensive care unit

Among the 59 ICU samples, 35 had bacterial culture reports. Out of 35, 26 (74.28%) were positive for the bacterial organism (mostly acinetobacter: 61.53%, 16/26). As shown in Figure 3, we further categorized the bacterial cultures (n = 26) isolated from the subjects based on their test results for various respiratory infections. It is noteworthy that patients with negative bacterial cultures also tested negative for the virus.

## Discussion

Recent developments in respiratory agent detection have led to many studies showing that some patients with acute lower respiratory tract infections also develop concurrent infections from multiple respiratory viruses [14]. The accurate and timely analysis of a variety of viral agents is essential for precise etiological investigation [12]. The goal of the analysis was to provide extensive information regarding viral causes in acute RTIs among adults in Bangladeshi populations. This study is the first to look into respiratory virus infections in adult ICU and OPD patients in the pre-COVID-19 era.

In this study, 49 specimens were positive for at least one respiratory virus. This study obtained a detection rate of respiratory viruses of 32.23%, comparable to that found by others [15,16]. HPIVs and HRVs (8.47% each) were the most frequently identified viruses, while HCoVs (10.78%) were prevalent in both community and hospital specimens. A pediatric study from Delhi found RSVs to be the most common (20.3% of 301 samples), with other viruses such as PIV1-3 and HMVs detected in 10-22 cases each. However, the study was done 15 years ago, and the children were younger than five at the time, so its relevance to the current study is limited [15].

Studies conducted among adults suffering from respiratory tract infections found that prevalence rates varied depending on the sample source; viruses were present more or less consistently across samples collected for testing. With 17.2% of cases, HRVs were found to be the most frequently detected virus, followed by respiratory syncytial virus B (15.4%), H1N1pdm09 IFV (8.544%), PIV3 (5.68%), and HMVs (5.2%) [16]. These findings suggest that respiratory viral infections are a major cause of respiratory tract infections in both adult and pediatric populations. Moreover, comparable rates for their detection rates were observed in the studies conducted by Bharaj et al. [15] and Branche et al. [16]. Comparison between community and hospital settings showed that human coronavirus had the highest viral prevalence rate among collected specimens from communities (10.78%). Human rhinovirus/parainfluenza virus infection rates (8.47%) were more frequent at hospitals [15]. Thus, the distribution of viral causes may depend on patient population size and healthcare setting.

Multiple viral infections are often linked with higher fever, longer hospital stays, higher antibiotic usage, and an increased risk of admission to the ICU. The effects depend on the co-infecting viruses [17]. Of the specimens, 4.6% had dual infections primarily consisting of HCoV and IAV co-viruses, while in positive specimens, co-infection rates for HCoV were higher than other respiratory viruses [18]. This study revealed a high frequency and severity of coronavirus infections in Bangladesh. Various factors might contribute to their prevalence and severity, such as demographic profile, health system capacity, public health measures taken against viral variants, and vaccine coverage [13].

The evidence of dual or multiple viral infections and viral-bacterial co-infection is also documented, and older adults with co-infection lead to increased disease severity [19]. Though the frequency determination of bacterial causes of ARTI was not an objective in this study, viral-bacterial co-infections were also documented in the collected respiratory samples. Nevertheless, no differences were observed in the clinical presentation or severity in terms of infection from viral or bacterial origin [19]. Viral presence in the respiratory epithelium is thought to predispose bacterial colonization by altering the mucosal surface [20]. However, viral pneumonia's severity and mortality rates are nearly comparable to bacterial pneumonia [21]. In patients with predisposing factors such as immunocompromised conditions or sepsis, the outcome may be fatal with the development of severe pneumonia or acute respiratory distress syndrome, which may require hospitalization in ICUs [22]. Considering all facts, this study might be one of the pioneers in observing the circulation of single and multiple respiratory viruses across various community- and hospital-admitted adult populations and co-detection with bacteria using a multiplex real-time PCR system, which could be the fastest laboratory technique to detect virus, and patients can be cured as early.

Despite several strengths of this study, it has some limitations such as the following: it has a small sample size, it is concise to a specific timeframe between December 2018 and April 2020, and multiplex real-time PCR assay was employed to target 16 respiratory viruses, which means that to prevent the interactions between viruses and bacteria that cause respiratory infections, more viruses than less common or emerging viruses are required. Though a European conformity (CE)- and in vitro diagnostics (IVD)-certified respiratory assay was employed in the current study with improved sensitivity and specificity, however, the potential for cross-reactivity and false positives linked to specific target sequences could not be completely ruled out. Additionally, the study's limitations in differentiating the direct impact of individual viruses in co-infection cases and the possible underrepresentation of certain viruses due to the specificity of the multiplex PCR assay complicate the analysis and generalizability of the findings.

The significance of this study lies in its comprehensive analysis of respiratory viral pathogens among patients with symptomatic respiratory infections in Dhaka City. By identifying the prevalence and distribution of various respiratory viruses, the study provides valuable baseline data that can inform clinical practice and public health strategies. This is particularly relevant for understanding the typical viral landscape in pre-pandemic conditions, which can be compared with post-pandemic trends. The study supports further research by establishing a foundation for exploring changes in viral patterns and infection dynamics in the post-pandemic era. It highlights the need for ongoing surveillance and research into emerging respiratory viruses and their impacts on different patient populations.

## Conclusions

This research identifies key viruses associated with acute respiratory infections in Bangladeshi adults. Moreover, it emphasizes that the multiplex PCR technique significantly accelerates virus detection and co-infection identification, facilitating more personalized treatment approaches and better patient outcomes.
